# Effectiveness of L-thyroxine treatment on TSH suppression during pregnancy in patients with a history of thyroid carcinoma after total thyroidectomy and radioiodine ablation

**DOI:** 10.2478/v10019-012-0003-5

**Published:** 2012-01-02

**Authors:** Blaz Krhin, Nikola Besic

**Affiliations:** 1 Department of Laboratory Diagnostics, Institute of Oncology Ljubljana, Ljubljana, Slovenia; 2 Department of Surgical Oncology, Institute of Oncology Ljubljana, Ljubljana, Slovenia

**Keywords:** pregnancy, TSH suppression, L-thyroxine, thyroid carcinoma

## Abstract

**Introduction.:**

There are scarce data about the optimal increase of L-thyroxine dose during pregnancy in patients with a history of thyroid carcinoma. The first aim of the study was to find out if routine therapeutic measures enable adequate TSH suppression in pregnancy. The other aim was to find out the optimal dose of L-thyroxine for TSH suppression in pregnant women.

**Patients and methods.:**

In this retrospective observational study, we analysed 36 pregnancies of 32 women with a history of thyroid carcinoma. Before pregnancy, all of them underwent total thyroidectomy and radioiodine ablation of thyroid remnant, and they were on suppressive doses of L-thyroxine. Thyroid function tests were obtained before, during and after pregnancy.

**Results:**

Mean L-thyroxine dose before pregnancy, in the first, second and, third trimester and after delivery was 149, 147, 155, 165 and 158 micrograms daily, respectively. TSH concentration remained suppressed in 9 pregnancies, it was within normal range in 22 and elevated in 5 pregnancies. The mean dose of L-thyroxine in patients with suppressed TSH before pregnancy, in the first, second and, third trimester and after delivery was 154, 154, 164, 160 and 161 micrograms daily, respectively. When the dose had to be changed, the mean increase of the dose was 31.5 micrograms daily.

**Conclusions:**

The range of changes in TSH concentration during pregnancy in the patients who have been on suppressive L-thyroxine therapy before conception is quite wide. TSH was adequately suppressed in only 25% of pregnancies. The dose of L-thyroxine in patients with suppressed TSH in the first, second and third trimester was 154, 164 and 160 micrograms daily, respectively.

## Introduction

Thyroid hormones are important for normal pregnancy and the foetal development.^1^,^2^ During pregnancy, maternal thyroid hormones requirements increase.^3^,^4^ It is well known that the reference values of TSH, free T_3_ and free T_4_ for healthy the non-pregnant population are not the same as during pregnancy. Dashe *et al*.^5^ published the data about the TSH concentration in 13,599 pregnancies. They found out that the normal physiological concentration of TSH during the first trimester of pregnancy was as low as 0.1 mU/L. According to the trimester-specific reference ranges of serum, TSH concentrations above 2.3 mU/L in the first trimester and 3.1–3.5 mU/L in the second and third trimester may already be indicative of subclinical hypothyroidism.^1^,^6^ Safety of pregnancy during subclinical hyperthyroidism was reported by Casey *et al*.^7^ They found out that the subclinical hyperthyroidism was not associated with the adverse outcome of pregnancy.^7^

The patients having undergone thyroidectomy and radioiodine therapy are dependent on exogenous L-thyroxine.^8^ Some authors advocate that the increase of dose of L-thyroxine during pregnancy should be determined from the results of thyroid function tests^8^–^11^, while others propose to increase L-thyroxine dose as soon as pregnancy is confirmed.^12^,^13^

There are very limited data in the literature about the changes of TSH and thyroid hormones during pregnancy in patients with a history of thyroid carcinoma. To our knowledge, these studies included only a small number of patients^8^, ^12^–^15^ with the largest study group of 18 such cases reported by Loh *et al*.^8^ There are scarce data about the optimal increase of L-thyroxine dose during pregnancy in the patients with thyroid carcinoma after total thyroidectomy and radioiodine ablation of thyroid remnant. The first aim of the study was to find out if routine therapeutic measures enable adequate TSH suppression in pregnancy. The other aim was to find out the optimal dose of L-thyroxine for TSH suppression in pregnant women.

## Material and methods

In this retrospective observational study we analysed 36 pregnancies of 32 women (mean age at conception 29.9 ± 0.6 years) with a history of thyroid carcinoma during the period from 2000 to 2009. T1, T2 and T3 tumour was diagnosed in 15, 7 and 10 patients, respectively. In 23 and 9 patients, the tumour stage was assessed as N0 and N1, respectively. None of the patients had distant metastases. All of them had no evidence of disease at conception and after delivery. Histopathology of bioptic specimen revealed the presence of Hashimoto’s thyroiditis in 11/36 (=31%) of patients. Before pregnancy, all of them underwent total thyroidectomy and radioiodine ablation of the thyroid remnant and they were all on suppressive doses of L-thyroxine. All patients were advised to take L-thyroxine four hours before vitamins, iron or calcium drugs.

At conception, the age of patients was 22 to 37 years (mean age 29.9 ± 0.6 years). Among our patients, 28 women were pregnant once and four twice. Pregnancy passed without any events in 30 cases and with complications in six cases (premature delivery in two cases, preeclampsia in one, increased blood pressure in one, high serum glucose concentration in two, vaginal bleeding in the seventh month of gestation in one case).

Thyroid function tests were performed before, during and after pregnancy. The last test of thyroid function before pregnancy was done 1–12 (median 3) months before conception. During pregnancy, a clinical exam and thyroid function tests were performed every 6–8 weeks. When more than one set of tests was performed in any one trimester, the highest TSH concentration with the corresponding free T_3_ and free T_4_ concentrations were used in the statistical analysis in order to minimize any bias towards avoidance of dose change. The median number of thyroid function tests performed during pregnancy in each woman was four (range3–6). L-thyroxine dose was modified to maintain serum TSH below 0.3 mU/L. Suppression dose was adequate if TSH was 0.01–0.29 mU/L and free T_3_ was within normal range.

From 2000–2006, TSH was measured by the two-site immunoluminometric assay (sandwich principle) LIASON TSH (Byk-Sangtec Diagnostica, Dietzenbach, Germany). Free T_3_ and free T_4_ were measured by commercially available kits (LIAISON FT_3_, LIAISON FT_4_) with “LIAISON” Immunoassay System (Byk-Sangtec, Germany later DiaSorin, Italy). Reference values for TSH, free T_3_ and free T_4_ were 0.27–4.2 mU/L, 2.93–6.8 pmol/L and 7.7–23.2 pmol/L, respectively. From 2007 onwards, TSH, free T_3_ and free T_4_ were measured by commercially available kits (TSH, FT_3_, FT_4_) with “Modular Analytics E170” Immunoassay System (Roche Diagnostics, Mannheim, Germany). Reference values for TSH, free T_3_ and free T_4_ were 0.27–4.20 mIU/L, 3.1–6.8 pmol/L and 12–22 pmol/L, respectively.

The study was reviewed by the appropriate medical ethics committee. The Institute’s Protocol Review Board approved the study, which was performed in accordance with the medical ethics standards laid down in an appropriate version of the 1964 Declaration of Helsinki. The participants gave informed consent.

### Statistical analysis

Changes in the results of thyroid-function tests, and L-thyroxine doses throughout pregnancy were analysed by repeated measures ANOVA or Friedman’s test, followed by Wilcoxon signed rank test in case of non-normal data distribution. P-values of less than 0.05 were considered to indicate statistical significance. The software package SPSS 16.0 for Windows (SPSS Inc., Chicago, IL USA) was used.

## Results

**FIGURE 1:**
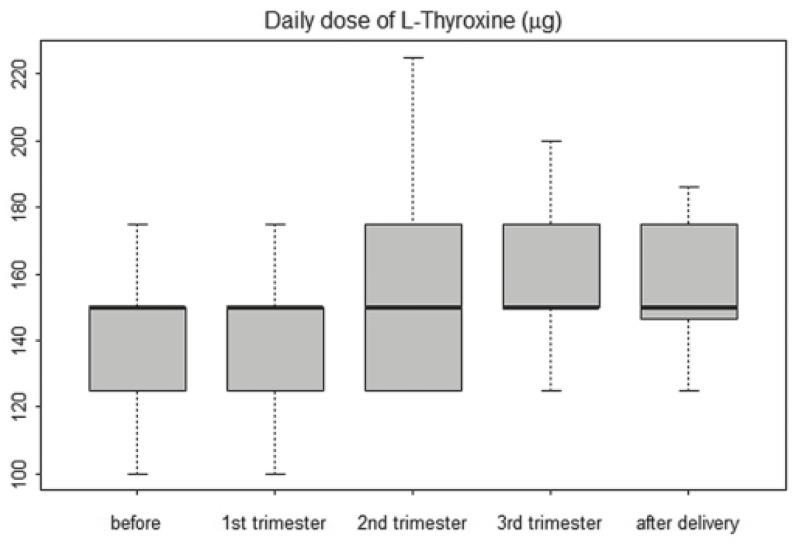
Changes of TSH during 36 pregnancies. The bottom and top of the box are the 25th and 75th percentile (the lower and upper quartiles, respectively), and the band near the middle of the box is the 50th percentile (the median). The ends of the whiskers represent the minimum and maximum of all the data.

The mean concentration of TSH, freeT_4_ and free T_3_ before pregnancy, in the first, second and third trimester and after delivery are presented in Table 1. The concentration of TSH during the first, second and third trimester were higher in comparison to the TSH concentration before pregnancy (p<0.001). The changes of TSH concentration during pregnancy are presented in Figure 1. In none of our patients, a decrease of TSH was observed in the first 16 weeks of pregnancy.

**FIGURE 2:**
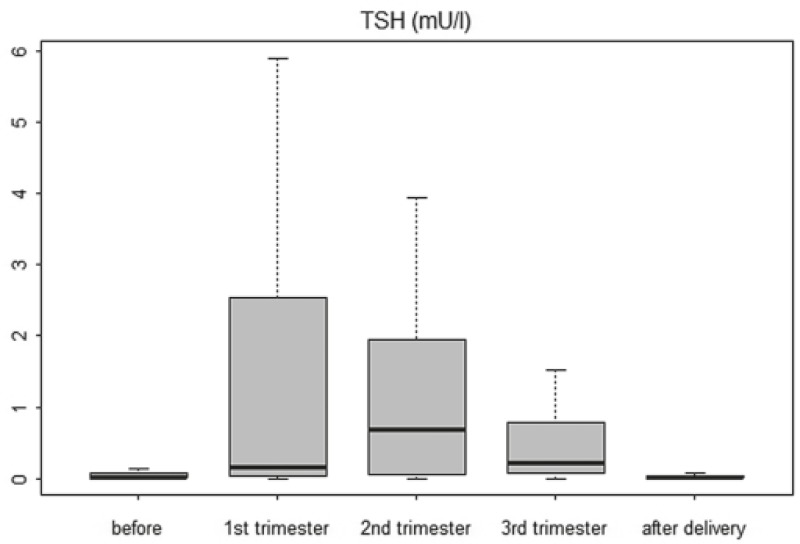
Changes of dose of L-thyroxine during 36 pregnancies. The bottom and top of the box are the 25th and 75th percentile (the lower and upper quartiles, respectively), and the band near the middle of the box is the 50th percentile (the median). The ends of the whiskers represent the minimum and maximum of all the data.

The doses of L-thyroxine during the second and the third trimester were higher than the doses before pregnancy (p<0.05). The changes of dose of L-thyroxine during pregnancy are presented in Figure 2. Obese patients had larger dose of L-thyroxine in comparison to patients with normal body mass index (BMI) or underweight patients before conception as well as during pregnancy. However, BMI had no impact on the proportion of patients with adequate TSH suppression during pregnancy. The time period from the last test before pregnancy to the first test in pregnancy did not differ in women with adequate TSH suppression and women with inadequate TSH suppression (p=0.377).

Changes of the mean TSH concentration and dose of L-thyroxine before and during pregnancy and after delivery in patients with suppressed and not suppressed TSH concentration are presented in Table 2. The mean dose of L-thyroxine in patients with suppressed TSH before pregnancy, in the first, second and, third trimester and after delivery was 154, 154, 164, 160 and 161 micrograms daily, respectively.

In 36 pregnancies, the TSH concentration remained suppressed during 9 pregnancies. In 22 pregnancies the TSH concentration was within normal range,whilein5 pregnancies it was elevated. A dose of L-thyroxine was not changed in 14 pregnancies (mean dose 159 micrograms daily); TSH was suppressed in 5 pregnancies and within normal range in 9 pregnancies.

The dose of L-thyroxine had to be increased in 22 pregnancies. The TSH concentration in 22 pregnancies remained suppressed in 4, within normal range in 13 and elevated in 5 of them. TSH was over 5.0 mU/L during the first trimester and second trimester in 5/22 (23%) cases and 3/22 (14%) cases, respectively.

The dose of L-thyroxine was increased 30 times and decreased four times during the course of pregnancy. The dose was changed 9, 11 and 14 times in the second, third and the fifth month, respectively. The mean increase of L-thyroxine dose in these 22 cases was 32.15 (range 25–75) micrograms.

## Discussion

According to the current American Thyroid Association and European Thyroid Association guidelines^16^,^17^, the patients with a history of thyroid carcinoma are either on substitution or suppressive doses of L-thyroxine. Our study group consisted of 36 pregnancies in 32 patients with a history of thyroid carcinoma. Before conception they were treated by total thyroidectomy and radioiodine ablation of thyroid remnant and were on the suppressive dose of L-thyroxine. So, in all of our patients there was no viable thyroid tissue to produce thyroid hormones. These patients were, therefore, entirely dependent on exogenous L-thyroxine.

In healthy women, a transient decrement of serum TSH concentration occurs during the first two months of pregnancy, which is a result of the elevation of human chorionic gonadotropin (hCG) having similar molecular structure as TSH.^1^,^18^,^19^ In none of our patients, lower concentration of TSH was detected during the first two months of pregnancy. In the absence of viable thyroid tissue, there was no effect of elevated concentration of hCG on the synthesis and secretion of thyroid hormones from thyroid and on the consequent decrease of TSH concentration.

According to Endocrine Society Clinical Practice Guideline^1^, L-thyroxine dose often needs to be increased within 4–6 weeks of gestation and may require a 30–50% addition of dosage.^3^,^4^ But we observed that there was no need to increase the dose during pregnancy in 25% of our cases who were on suppressive doses of L-thyroxine before conception. Also Loh *et al*.^8^ observed that there was no need to change the dosage in some of the patients with thyroid carcinoma who were on TSH suppression therapy before pregnancy. Based on our results, we believe that an increment of dose immediately after conception as advocated by some authors^12^ is not appropriate for all the patients who are on suppressive doses of L-thyroxine. We agree with Loh *at al*.^8^ that these patients may be over treated by an empiric increase of L-thyroxine dose and may develop overt hyperthyroidism.

The other important finding of our study is that TSH suppression was not achieved in 75% of cases when thyroid testing was performed each 6–8 weeks. Furthermore, an elevation of TSH level above normal was observed in 14% of our patients. Possibly, this could be avoided, if thyroid function tests would be obtained every 4–6 weeks as recommended by Endocrine Society Clinical Practice Guideline^1^, or every 4 weeks as recommended by Yassa *et al*..^13^ In a recent study Yassa *et al*.^13^ performed serum testing every two weeks until the 20^th^ week of pregnancy and at the 30^th^ week of pregnancy. If blood samples were obtained every four weeks, 92% of abnormal TSH concentrations were detected. But, if an every six week testing protocol had been followed, only 73% of abnormal TSH concentrations would have been detected.^10^ So, they concluded that the thyroid function tests should be repeated every four weeks during the first half of pregnancy.

It is still not known when it is most appropriate to increase the dose of L-thyroxine^20^: before pregnancy^21^, immediately after conception^12^,^13^ or when elevation of TSH is observed.^8^–^11^ Rotondi *et al*.^21^ suggested the increase of L-thyroxine dose to “partially suppressive” dose before pregnancy. The patients who underwent thyroidectomy because of multinodular goitre or Hashimoto’s thyroiditis and who were on substitution therapy were randomized in two groups before conception: one group continued with substitution doses, while the other was on partially suppressive dose.^21^ Before conception, the average dose in the substitution group and the partially suppressive group of patients was 143 and 178 micrograms, while the average TSH was 1.84 and 0.48 (0.32–0.7) mU/L, respectively.^21^ TSH level above 3.5 mU/L was found to be more common in the substitution group in comparison to the “partially suppressive” group of patients (36% vs. 14%).

Another approach, *i.e.* increment of the dose as soon as pregnancy is confirmed, was proposed by Alexander *et al*..^12^ They studied precisely the timing and amount of L-thyroxine adjustment in 20 pregnancies in 19 women.^12^ Eight patients had Hashimoto’s disease, six thyroid carcinoma, three Graves disease and two were after the treatment for benign thyroid nodule. L-thyroxine requirements increased as early as the fifth week of gestation.^12^ That is why they recommended an increase of L-thyroxine dose as soon as possible.^12^ Their opinion is that the dose should be increased by about 30%.^12^ Yassa *et al*.^13^ performed a prospectively randomized study in which women were receiving an increased L-thyroxine dose at the beginning of pregnancy. The dose in the first group was increased by 29%, and in the second group, by 43%. The mean dose before and after the change in the first and the second group was 112, 145, 109 and 156 micrograms, respectively.^13^ After the change of dose, TSH suppression was present in 32% of women from the first group and in 65% of women from the second group; but less than 0.1 mU/L of TSH was found in only one patient (8%) from the first group and in six patients from the second group (26%).^13^ They found out that a 29% incr ease of dose (from 112 to 145 micrograms) prevented maternal TSH elevation over 2.5 and 5.0 mU/L in 85% and 100% of patients, respectively. But the majority of their patients had Hashimoto’s disease, while our patients had a history of thyroid carcinoma and were without any functional thyroid tissue. In our patients, the mean dose before pregnancy was 149 micrograms of L-thyroxine. When a dosage during pregnancy remained the same, the mean dose was 159 micrograms. This dosage prevented the elevation of TSH over 5.0 mU/L in all the cases. On the other hand, when a dosage during pregnancy had to be changed, the mean dose before conception was 125 micrograms only. In the latter cases, TSH was over 5.0 mU/L during the first and second trimester in 23% of cases and 14%, respectively.

The third approach, increment of a dose based on thyroid function tests was reported by Loh *et al*..^8^ In 18 cases with a history of thyroid carcinoma, the average daily L-thyroxine dose before conception was 153 micrograms. During pregnancy they required an increase of dose by 26%.^8^ In our patients, the average dose was 149 micrograms before conception. But during pregnancy our patients required an increase of dose by 11% only. Obviously the range of changes in TSH concentration during pregnancy in the patients who have been on suppressive L-thyroxine therapy before conception is quite wide.

As a conclusion, the patients with thyroid carcinoma who are on high doses of L-thyroxine require close monitoring of thyroid function tests during pregnancy. TSH was adequately suppressed in only 25% of pregnancies. The dose of L-thyroxine in patients with suppressed TSH in the first, second and third trimester was 154, 164 and 160 micrograms daily, respectively. When the dose had to be changed, the mean increase of the dose was 31.5 (range 25–75) micrograms daily.

## Figures and Tables

**TABLE 1:** Mean L-thyroxine dose and mean TSH concentration before pregnancy, in the first trimester, the second trimester, the third trimester and after delivery.

	**Daily dose of L-thyroxine Mean (±SD; range) Micrograms**	**Mean TSH (±SD; range) mU/L**	**FT4 (±SD; range) pmol/L**	**FT3 (±SD; range) pmol/L**
Before pregnancy	149 (±32; 100–300)	0.07 (±0.12; 0.001–0.29)	20.13 (±5.08; 13.9–28.4)	5.16 (±0.86; 3.91–6.61)
1. trimester	147 (±34; 100–300)	1.96 (±3.60; 0.001–14.54)	16.72 (±4.32; 9.29–27.6)	4.15 (±1.08; 2.41–7.6)
2. trimester	155 (±35; 125–300)	1.43 (±1.96; 0.001–7.64)	14.79 (±3.39; 8.95–24.1)	4.00 (±0.85; 2.72–5.5)
3. trimester	165 (±34; 125–300)	0.63 (±0.95; 0.001–4.60)	14.04 (±3.15; 5.07–23.1)	3.83 (±0.69; 2.52–5.2)
After pregnancy	158 (±34; 100–300)	0.05 (± 0.11; 0.001–0.59)	22.15 (±3.94; 14.6–31.9)	5.41 (±1.08; 3.18–7.83)

**TABLE 2:** Adequacy of suppression, mean L-thyroxine dose and mean TSH concentration before pregnancy, in the first trimester, the second trimester, the third trimester and after delivery.

		**Before pregnancy**	**First trimester**	**Second trimester**	**Third trimester**	**After pregnancy**
**Adequatelly supressed**	**Daily dose of L-thyroxine Mean (±SD) Micrograms**	154 (±32)	154 (±28)	164 (±47)	160 (±21)	161 (±33)
	**Daily dose of L-thyroxine/kg Mean (±SD) Micrograms/kg**	2.37 (±0.31)	-	-	2.17 (±0.30)	-
	**Daily dose of L-thyroxine/BMI Mean (±SD) Micrograms/kg/m2**	6.64 (±0.90)			6.03 (±0.80)	
	**Mean TSH (±SD) mU/L**	0.049 (±0.07)	0.07 (±0.07)	0.057 (±0.07)	0.12 (±0.08)	0.03 (±0.06)
	**Number of patients**	34	21	12	22	34

**Not adequatelly supressed**	**Daily dose of L-thyroxine Mean (±SD) Micrograms**	125 (±0)	154 (±28)	152 (±27)	173 (±48)	112 (±18)
	**Daily dose of L-thyroxine/kg Mean (±SD) Micrograms/kg**	1.88 (±0.50)			2.35 (±0.36)	
	**Daily dose of L-thyroxine/BMI Mean (±SD) Micrograms/kg/m2**	5.13 (±1.46)			6.00 (±0.90)	
	**Mean TSH (±SD) mU/L**	0.5 (±0.21)	4.6 (±4.4)	2.2 (±2.08)	1.43 (±1.13)	0.49 (±0.14)
	**Number of patients (% of patients with TSH over 5 mU/L)**	2 (0%)	15 (33%)	24 (8%)	14 (0%)	2 (0%)
